# A Comparative Study of Salter Versus Pemberton Osteotomy in Open Reduction of Developmental Dysplastic Hips and Clinical Evaluation on Bhatti’s Functional Score System

**DOI:** 10.7759/cureus.12626

**Published:** 2021-01-11

**Authors:** Anisuddin Bhatti, Imamuddin Abbasi, Zeehan Naeem, Kiran Jaffri, Muhammad Yousuf Bhatti

**Affiliations:** 1 Department of Orthopaedics, Dr. Ziauddin Hospital, Karachi, PAK; 2 Department of Orthopaedics, Neurospinal & Cancer Care Institute (NCCI), Karachi, PAK; 3 Orthopaedic Surgery, Jinnah Postgraguate Medical Centre, Karachi, PAK; 4 Orthopaedic Surgery, Jinnah Postgraduate Medical Centre, Karachi, PAK; 5 Orthopaedic Surgery, Lyari General Teaching Hospital, Karachi, PAK

**Keywords:** acetabular index, bhatti’s functional score, developmental dysplastic hips, pemberton acetabuloplasty, salter osteotomy

## Abstract

Objective

The study was aimed to compare the outcome of Salter’s and Pemberton’s osteotomy to achieve adequate acetabular coverage in the open reduction of developmental dysplastic hips (DDH). The functional outcome was evaluated as measured on Bhatti’s Functional Score System (BFSS).

Patients and methods

The study includes 60 children with 82 hips of developmental dysplasia in walking-age children. They were operated on for open reduction and Salter’s or Pemberton’s pelvic osteotomy to achieve concentric anatomical reduction with good anterolateral coverage of the femoral head. Patients for Salter’s and Pemberton's osteotomies were randomly selected. Preference was given to Pemberton’s osteotomy in cases with double/irregular acetabulum and in bilateral DDH. All patients were operated on by a single surgeon from January 2014 to December 2016 and were followed up till June 2020. The overall radiological outcome was assessed on Severin’s classification, comparing the pre and postoperative acetabular index (AI) and the clinical outcome on Bhatti’s Functional Score System.

Results

The overall functional behavior on Bhatti’s Functional Scoring revealed satisfactory outcome (excellent and good) in 73.17% (60/82) hips. On the radiological evaluation, 85.36% (70/82) hips achieved satisfactory development of hips (Severin Class IAB and IIAB) while 12.19% (10) hips developed a moderate deformity of the hip (Severin Class III; p>0.05). Comparing outcomes in both the Pemberton and Salter groups, the acetabular index significantly reduced after both procedures (p<0.05), however, the Pemberton group was more effective than the Salter group. Avascular necrosis (AVN) of Caput Femoris was noticed in 9.57% (8) hips, subluxations in 2.43% (2) hips, and impingement and stiffness in 12.19% (10) hips. Salter’s group had more numbers of AVN and subluxations as compared to the Pemberton group, whereas impingement and stiffness were more in Pemberton’s but none in the Salter group.

Conclusion

The hips with Pemberton’s acetabuloplasty exhibited better acetabular coverage and progressive development of hips as compared to Salter’s osteotomy group. Both groups, however, behaved equally on functional assessment with Bhatti’s Functional Score System. The risk of subluxation and AVN was found higher in Salter's group, and femoroacetabular impingement in Pemberton’s group. Pemberton’s osteotomy was the best option for a single-stage open reduction in bilateral DDH in terms of less risk of bleeding, good stability, better postoperative pain control, and a second surgery to remove transfixation K-wires.

## Introduction

The satisfactory outcome of the open reduction in the developmental dysplastic hips (DDH) depends on strict adherence to a good initial reduction (i.e., intact Shenton’s line or <12 mm distance between the femoral head and the teardrop of the acetabulum) and immobilization for a minimum of six weeks in a spica cast. That allows the best possible early weight-bearing soon after the removal of cast and facilitates osseous remodeling of the dysplastic hip [1-4].

To achieve the best possible anterolateral coverage of caput femoris in walking age children, pelvic osteotomy becomes necessary in the majority; this includes Salter innominate osteotomy (SIO), Pemberton pericapsular osteotomy (PPO), Dega, and triple pelvic osteotomy as per age group, obtusity of the acetabulum, and surgeon’s judgment on Catterall’s test of stability [4]. The SIO redirects the entire acetabulum following a complete trans-iliac osteotomy. While triple pelvic osteotomy (Steel, Wenger, and Pol Le Coeur) combines innominate osteotomy with osteotomies of pubic and ischial rami. Both these re-directional osteotomies tilt the acetabulum in retroversion, improving the anterior and lateral coverage but reducing posterior coverage [2,5-6]. The PPO is an incomplete pericapsular osteotomy of the ilium. The anterolateral table of the ilium is displaced laterally. This improves anterolateral coverage and reduces the diameter of the acetabulum while the posterior coverage remains unchanged [5-7]. The Dega acetabuloplasty is again an incomplete transiliac pericapsular osteotomy, where the outer cortex is displaced laterally. Here, the inner table of the pelvis and the entire cortex of the sciatic notch is preserved. This acetabuloplasty reduces the diameter of the acetabulum and improves overall coverage of the caput femoris in all directions, anterior, lateral, and posterior [5-7]. The goals of these pelvic osteotomies are to improve the containment of the femoral head, facilitate early weight-bearing to allow osseous remodeling of the dysplastic acetabulum by the mutual growth-stimulating effect of the caput femoris on the acetabulum, and to minimize the risk of significant complications [1-2,5,8-10].

The PPO, despite having a high rate of successful results of better anterior coverage, may exhibit a higher risk of anterior impingement and avascular necrosis (AVN) of the caput femoris as compared to SIO [1-2,8-9,11]. The limitations with SIO include minimum correction of the acetabular index (AI) (i.e. <15 degrees) and the need for an additional surgical step to remove the trans-fixation wires [1-2,6,12-14]. Douglas and Lequesne implied it as a cause of hip pain, impingement, and subsequent osteoarthritis [1,15], whereas Wang found no difference in clinical outcome between the two procedures [2].

This study is planned to know the comparative effect of correction in the acetabular index with SIO versus PPO and to determine whether the anterolateral coverage achieved by these two osteotomies had any effect on the clinical outcome. Bhatti’s Functional Scoring System (BFSS) was used to evaluate the functional limitations of patients: impingement and discomfort in performing daily accustomed sitting habits [16]. Radiological assessment was made based on the Severin classification and comparing the pre versus the postoperative acetabular index [4,6,11-13].

## Materials and methods

This prospective, analytic, descriptive study includes 60 children (82 hips) of the walking age group having DDH, with Tonnis height of dislocation stages III and IV [4]. Patients were included irrespective of age, sex, and laterality. The patients with hip dislocations that mimic DDH due to paralytic, post-infective, teratologic, and post-trauma were excluded. The patients in the age group less than a year, above 12 years, and with a follow-up duration of less than three years were also excluded (Table 1).

**Table 1:** Basic demographics: age, sex, and lateral distribution (N-60 patients)

Particular	Female	Male	Grand Total		%
Bilateral	18	4	22		37%
1 ~ 3 Years	10	3	13		
3.1 ~ 5 Years	5	1	6		
5.1 ~ 8 Years	2	0	2		
Above 8 Years	1	0	1		
Unilateral (33% Right, 30% Left)	32	6	38		63%%
1 ~ 3 Years	20	3	23		
3.1 ~ 5 Years	5	1	6		
5.1 ~ 8 Years	4	1	5		
Above 8 Years	3	1	4		
Grand Total	50	10	60		100%

All the patients were operated on by a single surgeon (corresponding author), from January 2014 to December 2016, at Neurospinal and Cancer Care Institute (NCCI) and Jinnah Postgraduate Medical Centre, Karachi, Pakistan. They were further followed up till December 2019. The study started with the approval of the Institutional Review Committee and with informed consent from parents for inclusion in the study and the publication of photographs and videos taken.

**Figure 1:**
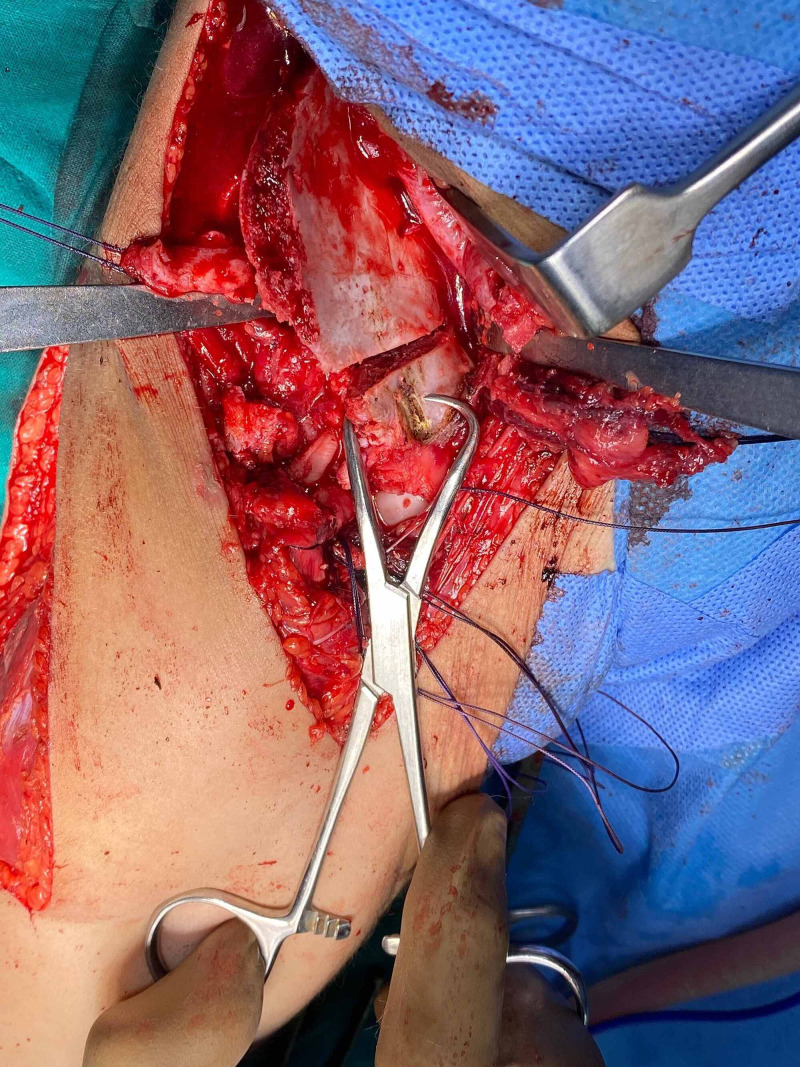
Salter's transiliac innominate osteotomy. Osteotomy directed at sciatic notch

**Figure 2:**
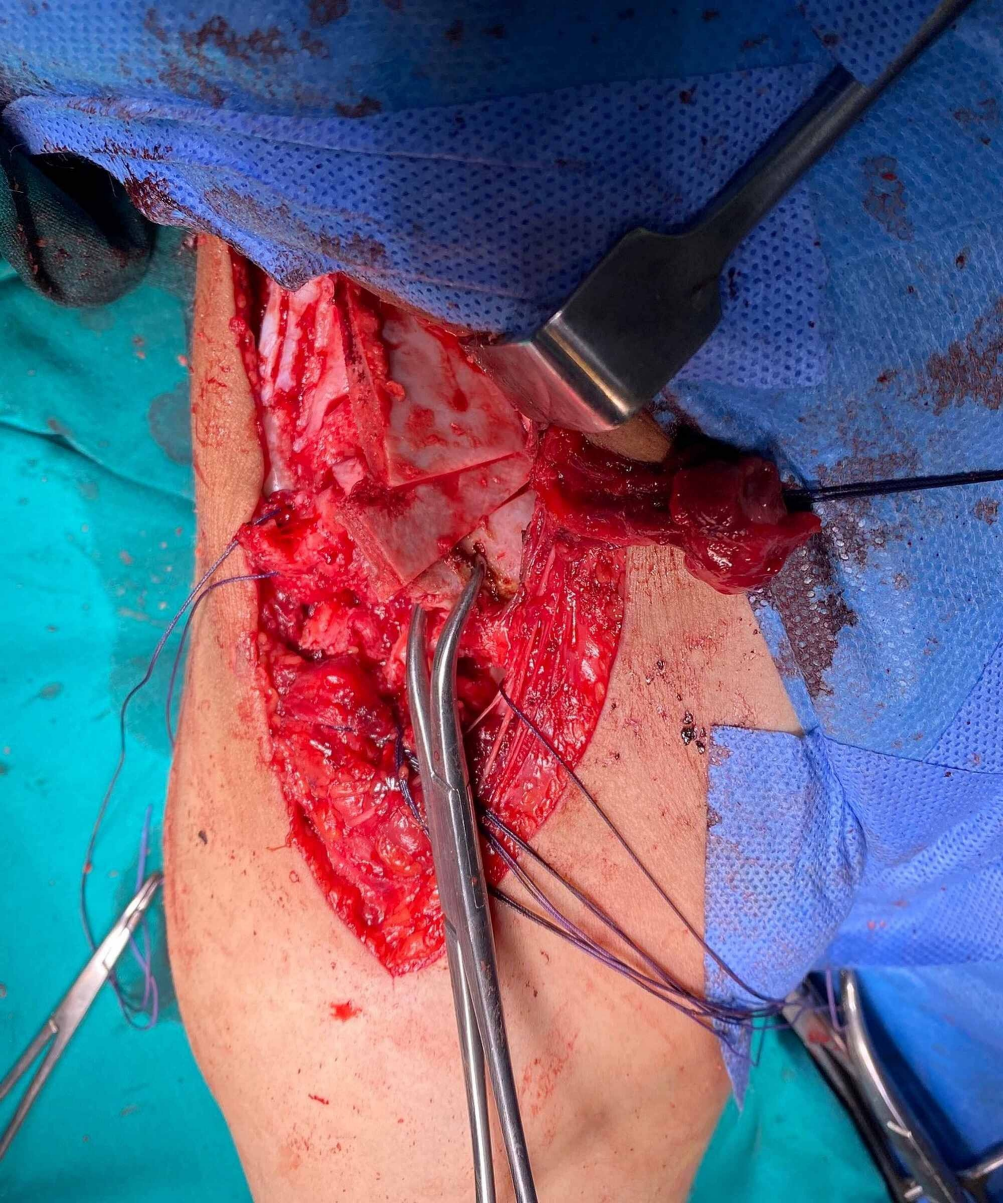
Salters's innominate osteotomy wedge-shaped graft in place

**Figure 3:**
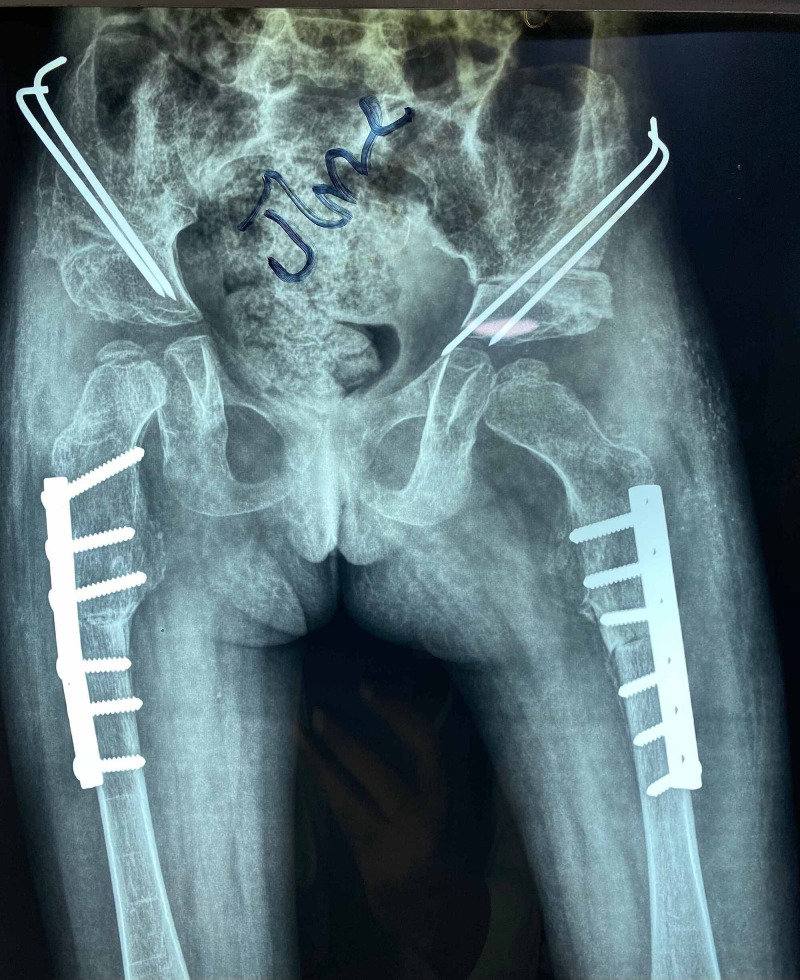
Salters innominate osteotomy graft in place and stabilized with K wires

**Figure 4:**
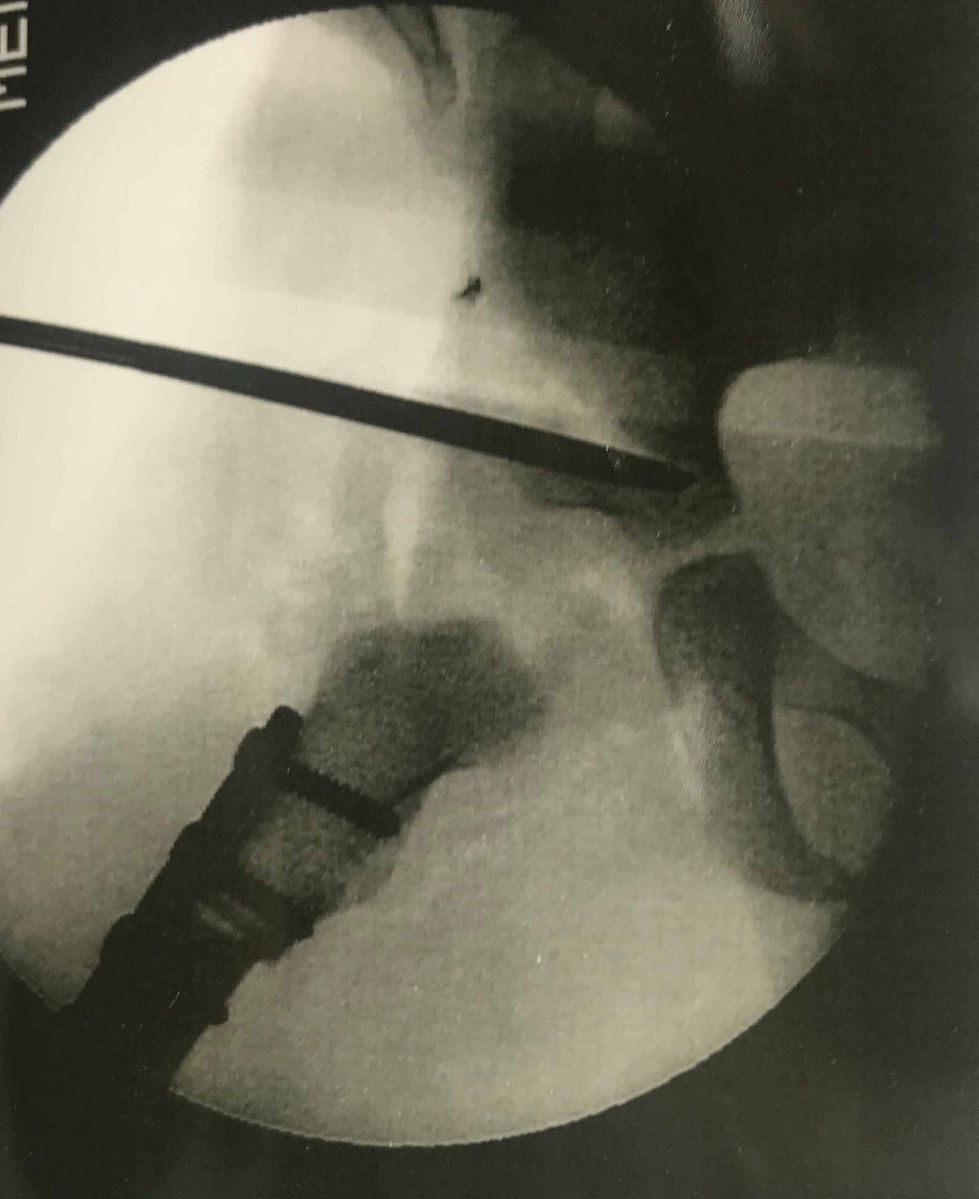
Pemberton periacetabular osteotomy. Osteotomy directed to triradiate cartilage

**Figure 5:**
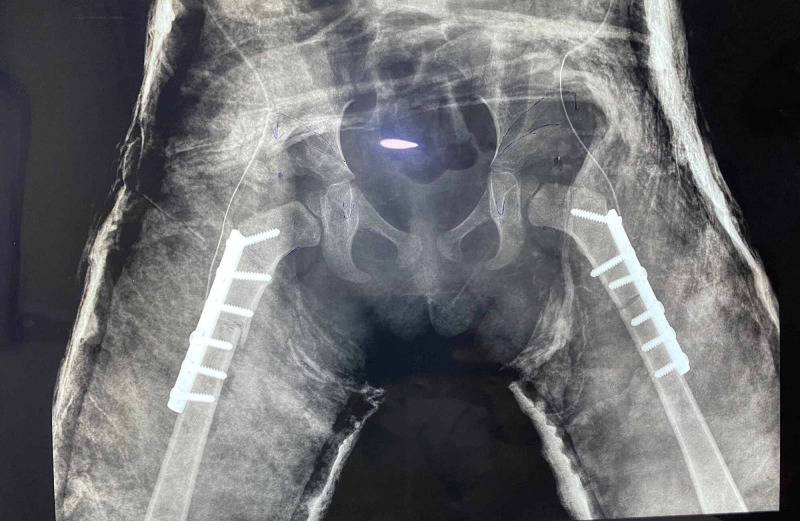
Pemberton graft in place in bilateral DDH DDH: developmental dysplastic hips

**Figure 6:**
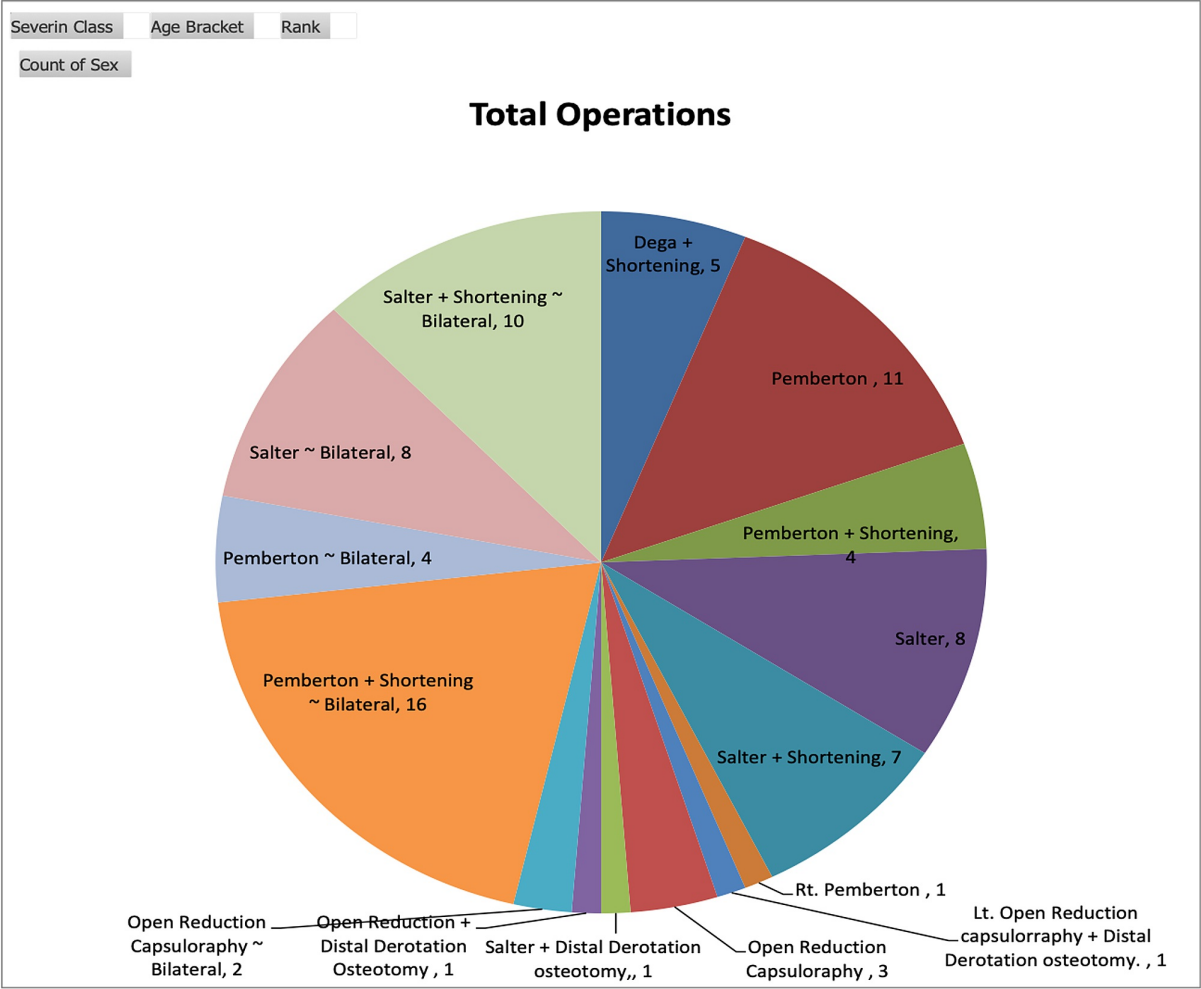
Total operative procedures (n=82)

Following a standard preoperative workup for general anesthesia, all the patients underwent anterior open reduction and capsulorrhaphy through an Ilio-femoral Smith Petersen approach [6]. The additional procedure of an SIO or PPO was performed on randomly selected patients (Figures 1-5). However, preference was given to PPO in DDH with double acetabulum and in bilateral DDH, facilitated further with Catterall’s test of stability [4]. The femoral shortening-derotation osteotomy in patients aged over 30 months was made through a lateral letterbox approach [7,13-14]. The bilateral hips under eight years were operated in a single go under the same setting. Patients younger than two years (nine hips) were operated on with open reduction and capsulorrhaphy without pelvic osteotomy. Dega’s acetabuloplasty was made in five hips of age group over five years (Figure 6). These cases of open reduction without pelvic osteotomy and Degas osteotomy were included to complete the quorum of DDH cases operated on during the study duration. Postoperatively, all the patients were immobilized with a hip spica cast for eight to 10 weeks. K-wires were used only in Salter’s osteotomy and were removed at the removal of the cast.

Clinico-radiological evaluations were made after the removal of the cast; thereafter, quarterly and at the most recent follow-up, i.e. not less than three years. The clinical outcome was evaluated on Bhatti’s Functional Scoring System (BFSS) [16]. The BFSS exhibits the patient’s limitations, discomfort, and impingement, if any, while performing the daily accustomed sitting habits of the Asian community (Table 2). The patient was asked to sit in the following positions: (i) squat (crouch sitting), (ii) *palthi* (cross-legged sitting), and (iii) *tashahhud* (kneeling or sitting on buttocks with kneeling). While performing these tests, a photograph was taken and a video was recorded as well. Each sitting habit was categorized as Type I, II, or III depending on limitations on sitting. These types are then grouped as excellent to poor. The patient’s score is indicated as excellent with a combination of SI + PI +TI; as good on combination when any one type of S, P, or T is in a Type II component; fair when all types are in Type II and/or one of these have a Type III component; and poor when all habits have the Type III category [16].

**Table 2:** Bhatti's Functional Scoring System Source [16]

Bhatti’s Functional Score System (BFSS)						
Sitting Habit	Type I		Type II		Type III	
Squat (S)	Able to squat comfortably		Able to squat with the heel raised, need support, feel discomfort		Unable to squat	
Palthi (P)	Able to make Palthi comfortably by touching knee to the floor		Able to make Palthi with knee raised from the floor for < 45^0^,needs support and feels discomfort		Unable to make palthi, knee raised from the floor over 45^0^	
Tashahhud (T)	Able to sit in Tashahhud easily.		Difficult to sit in tashahhud on the floor, feel discomfort. Easy on the chair with leg dropped down		Unable to sit in tashahhud on the floor or chair with the leg dropped down	
Score	Excellent	Good		Fair		Poor
	SI PI TI	SI PII TI, SII PI TI		SII PII TI, SII PII TII, SII PIII TII		SIII PIII TII, SIII PIII TIII

The overall radiological outcome of surgery was evaluated to know the progressive development of the hip on Severin classification [9,12-13]. The improvement in coverage of the caput femoris was assessed with a comparison of the pre-post-operative acetabular index (AI). We used the average acetabular index 22degree, which ranged from 15-25 degrees as a baseline comparative indicator, as was assessed in the normal side of our 38 unilateral DDH. That range of the acetabular index is within the AI range cited by Ibrahim et al. [17].

The hypothesis questions include if there was a greater advantage of PPO osteotomy and its superiority over SIO in terms of achieving acetabular anterior coverage without significant complications. And to evaluate the functional limitations of patients as measured on Bhatti’s Functional Score System [16].

The Statistical Package for the Social Sciences (SPSS) version 23 (IBM Corp., Armonk, NY) was used to analyze data. Mean and SD were computed for numeric variables while frequency and percentage were reported for categorical variables. Pre and post-change in the acetabular index were applied to assess the significant difference after procedures. Fisher’s exact test was applied to assess the significant difference between outcome and treatment, Severin class, age, gender, and Tonnis grade. P<0.05 was taken as statistically significant.

## Results

The basic demographics of registered patients, as cited in Table 1, reveals a preponderance of unilateral hips, i.e. 63% (38 patients), female gender, i.e 83% (50 patients), and age group less than three years, i.e. 60% (36 patients). The majority (96.34%; 79/82 hips) had a Tonnis height of dislocation stage IV. The majority (59%; 13/22 patients) of bilateral DDH were of one to three years of age and 27% (6/22) were in the three to five years' age group. The frequency of all operative procedures is displayed in Figure 6. That reveals a nearly equal frequency of the Salter and Pemberton groups. The remaining 14/82 (17.07%) hips were operated on with open reduction, with or without derotation femoral osteotomy and Dega’s pericapsular osteotomy. All the patients of the Salter and Pemberton group were of age two to eight years, open reduction without pelvic osteotomy in <2 years age, and Dega’s among 10-12 years.

The overall functional behavior of patients on Bhatti’s Functional Scoring at the minimum three years follow-up (Table 3) revealed a satisfactory (excellent and good) outcome in the majority 73.17% (60/82) hips and fair in 21.95% (18/82) hips. The satisfactory outcome of unilateral DDH was better than bilateral, i.e. 76.31% versus 70.45% hips. Similar was the status in the fair and poor rankings. On Fisher’s exact test, a statistically significant difference was found between the treatment groups and functional outcomes (p=0.001). The intragroup and intergroup outcome of age at the time of surgery versus the BFSS score reveals that the excellent to good results seem to decline as the age increased over two years. On Fisher’s exact test, a statistically significant difference was observed in the frequency of outcome on BFSS with respect to age (p=0.002). While statistically, no association was found between outcome, gender, and Tonnis grade (p>0.05).

**Table 3:** Comparison of age and laterality with outcomes on Bhatti’s Functional Score (n=82)

	Bhatti’s scoring system				p-value
	Poor	Fair	Good	Excellent	
Age groups					
1-3 years	2	5	14	28	
3.1-5 years	0	4	3	11	
5.1-8 years	2	4	1	2	0.002
>8 years	0	5	0	1	
Laterality					
Unilateral	2	9	6	21	0.628
Bilateral	2	9	12	21	

On intra-group observation, the outcome of the Salter and Pemberton groups on BFSS (Table 4) indicates that the Salter group behaved better (satisfactory outcome), with a 10% edge over the Pemberton group. Whereas both groups ranked fair in a nearly equal ratio and none ranked poor in the Salter group, on comparing the outcome of intergroup observations, the Salter’s group again behaved better, with a 3.32% edge over Pemberton’s.

**Table 4:** Comparison of treatment procedures and outcome on Bhatti's Functional Scoring DDH: developmental dysplastic hips

	Bhatti's Functional Scoring					
Treatment Procedure	Excellent	Good	Fair	Poor	Grand Total	p-value
Bilateral DDH (n=44 Hips)						
Salter + Shortening	9	1	-	-	10	0.001
Salter	4	3	1		8	
Pemberton + Shortening	8	2	4	2	16	
Pemberton	-	3	2	-	5	
Open Reduction + Capsuloraphy	-	3	-	-	3	
Dega + Shortening	-	-	2	-	2	
Unilateral DDH (n=38 Hips)						
Salter	6	1	1		8	0.001
Salter + Shortening	2	1	4	-	7	
Pemberton	8	2	-	1	11	
Pemberton + Shortening	2	1	-	1	4	
Open Reduction + Capsulorrhaphy	3	1	-	-	4	
Dega + Shortening	-	-	3	-	3	
Grand Total	42	18	18	4	82	

**Figure 7:**
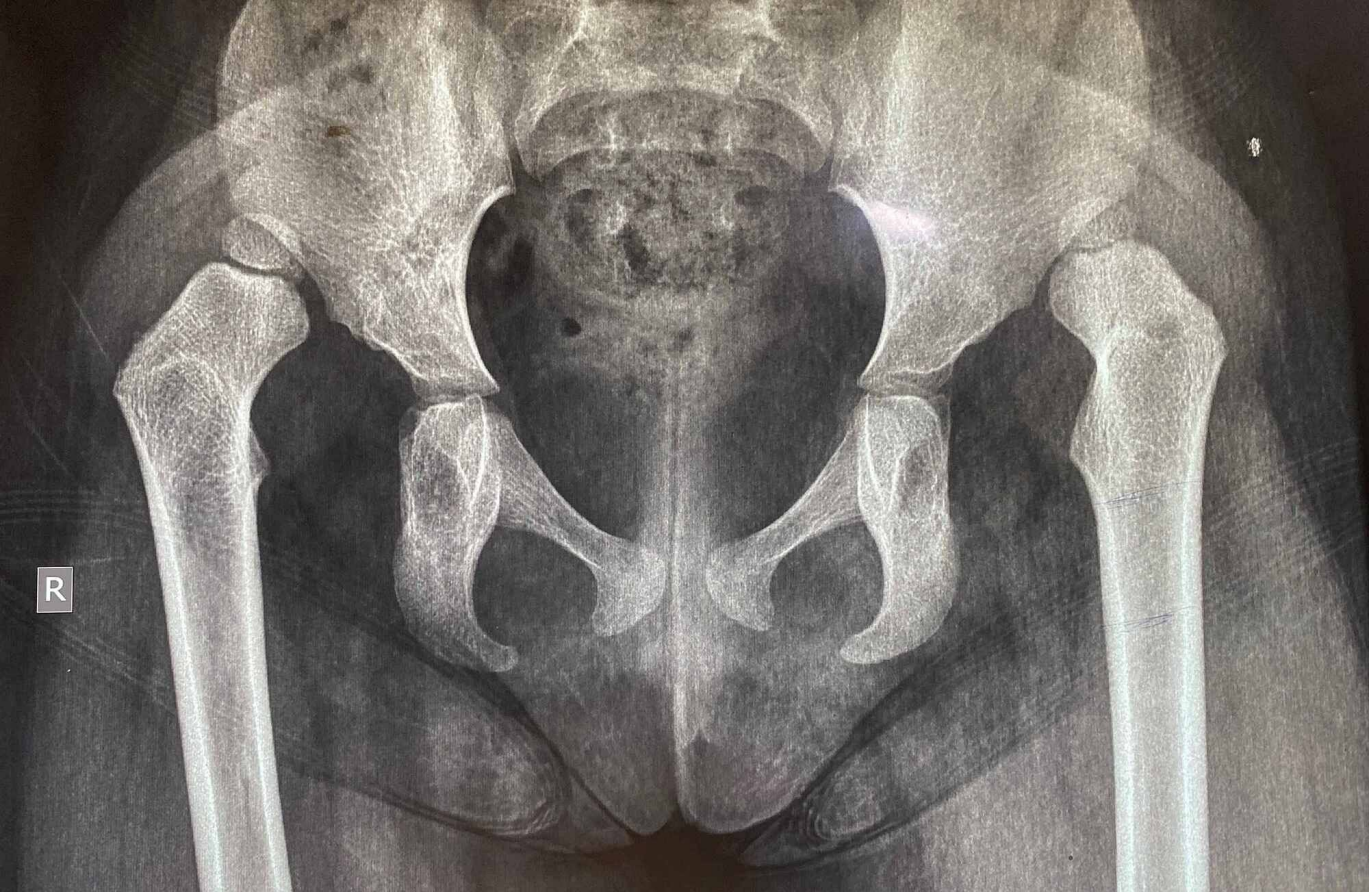
The X-ray of a three-year-old girl with bilateral DDH, Tonnis height of dislocation stage IV, both hips operated simultaneously DDH: developmental dysplastic hips

**Figure 8:**
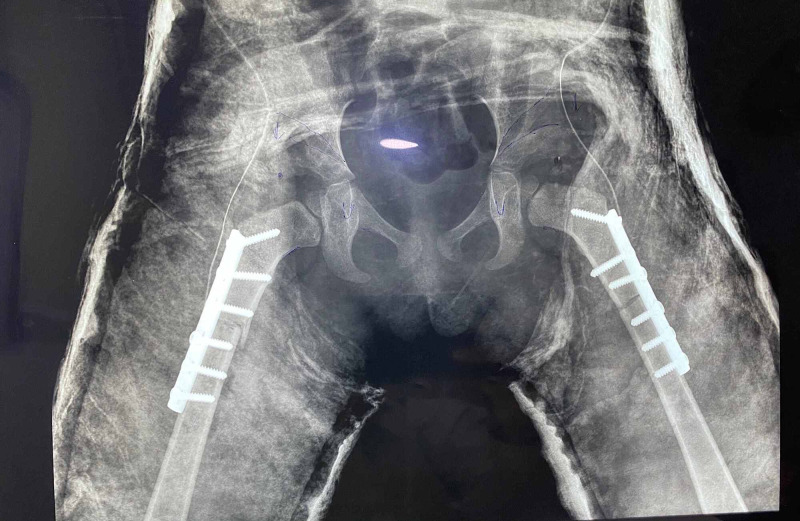
Bilateral Pemberton acetabuloplasty with femoral shortening derotation, both hips operated simultaneously

**Figure 9:**
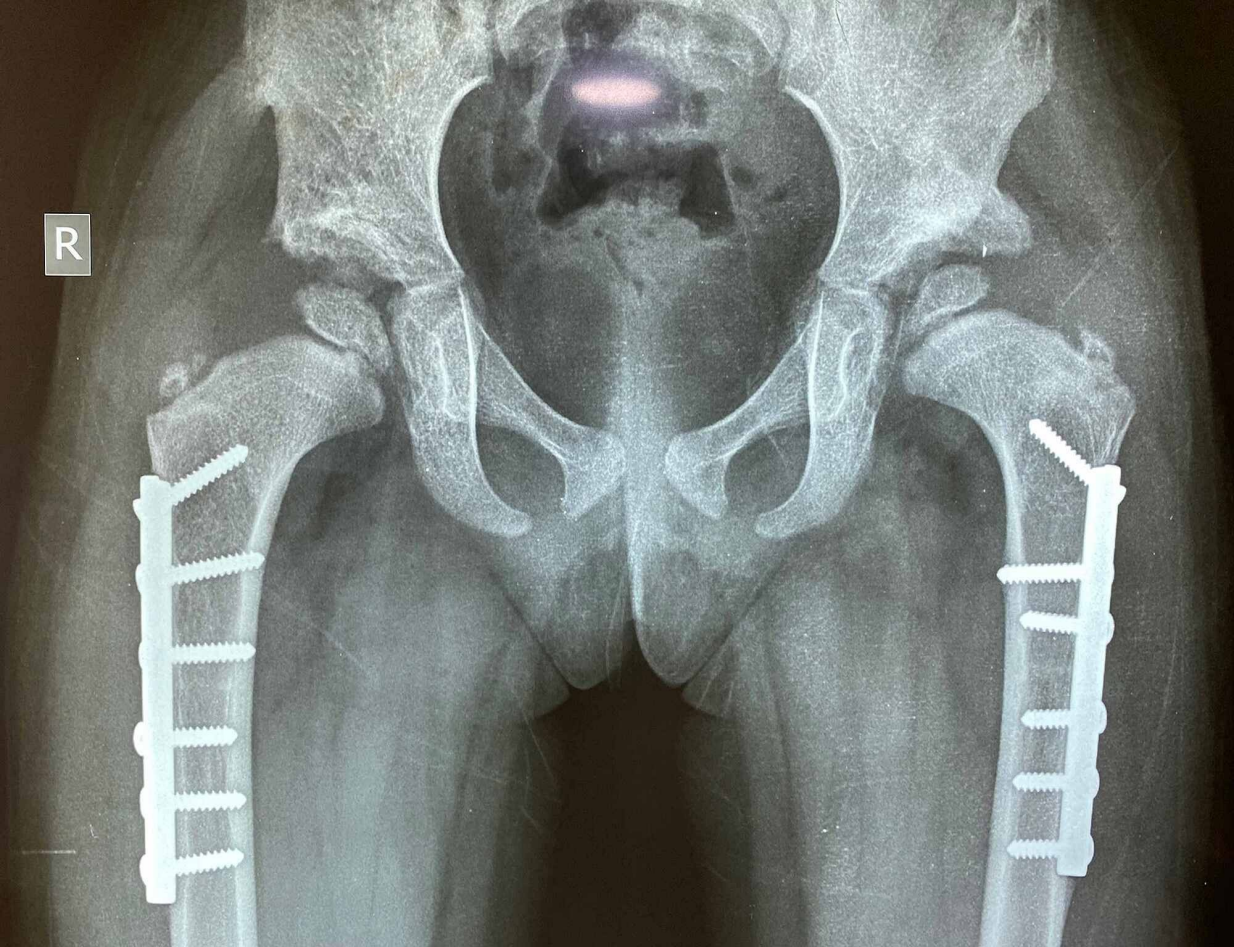
Bilateral Pemberton acetabuloplasty at three years' follow-up; well-developed hip; Severin Class I

**Figure 10:**
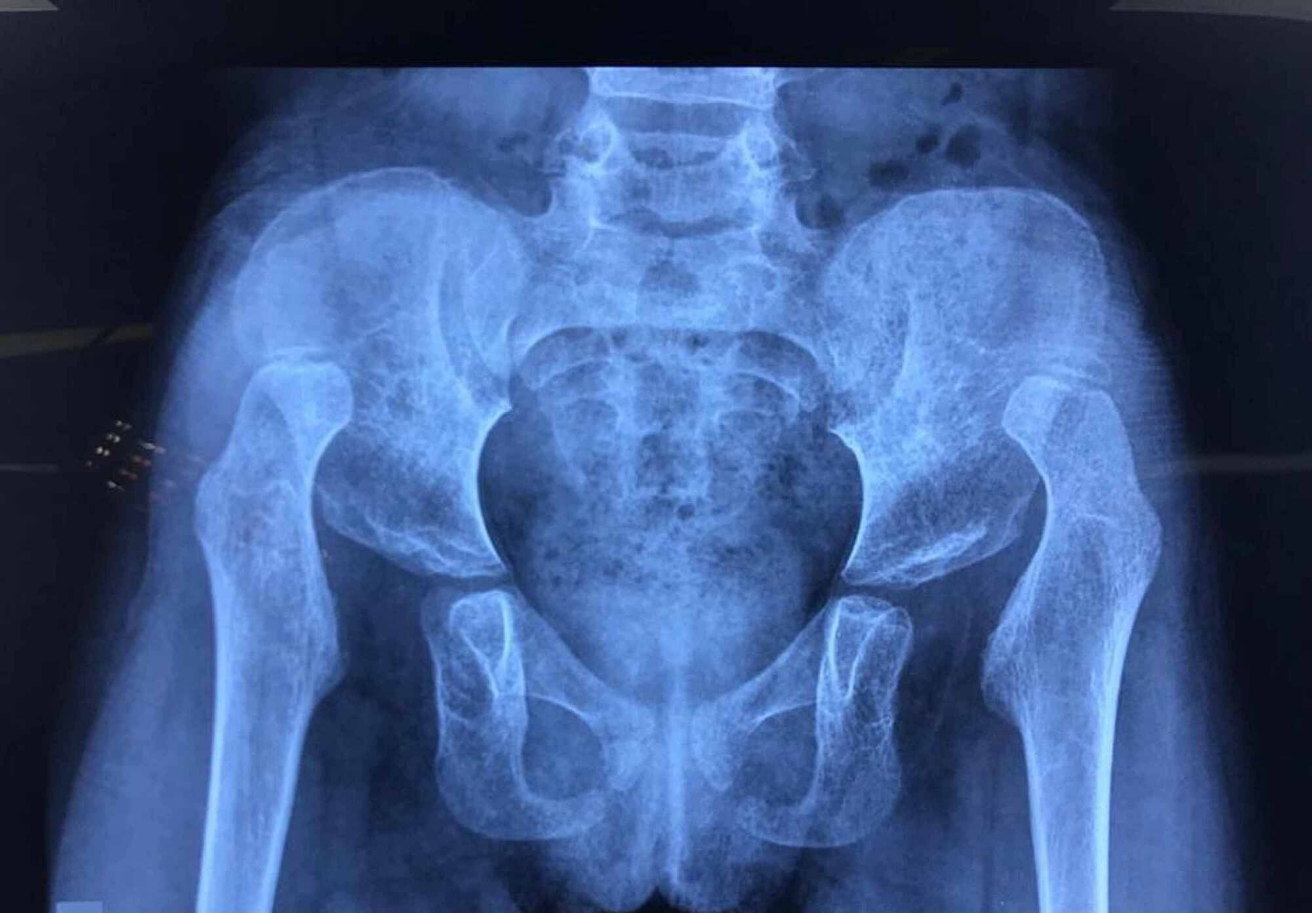
X-ray of a three-year and six-month-old girl with bilateral DDH, Tonnis stage IV; both hips operated simultaneously DDH: developmental dysplastic hips

**Figure 11:**
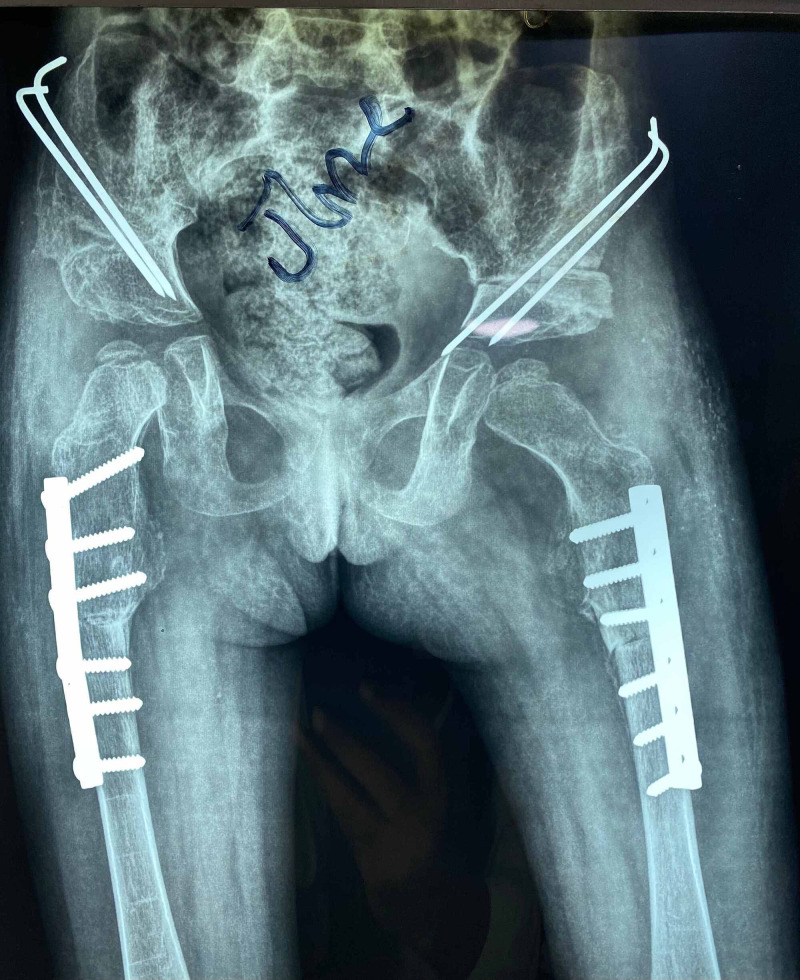
Post Salter's innominate osteotomy with graft in place and stabilization with K wires

**Figure 12:**
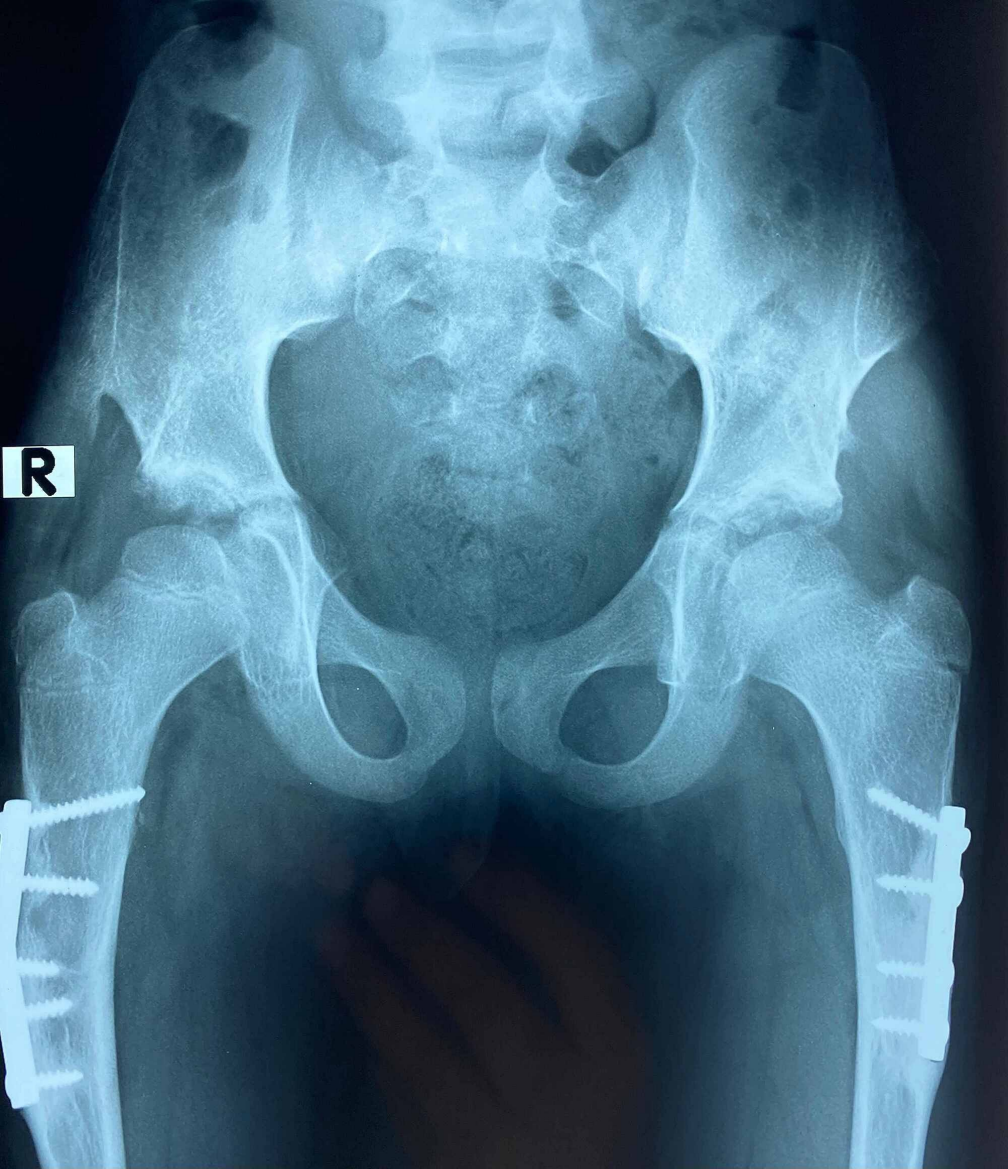
Post Salter's innominate osteotomy, three years' follow-up; Severin Class I outcome

**Figure 13:**
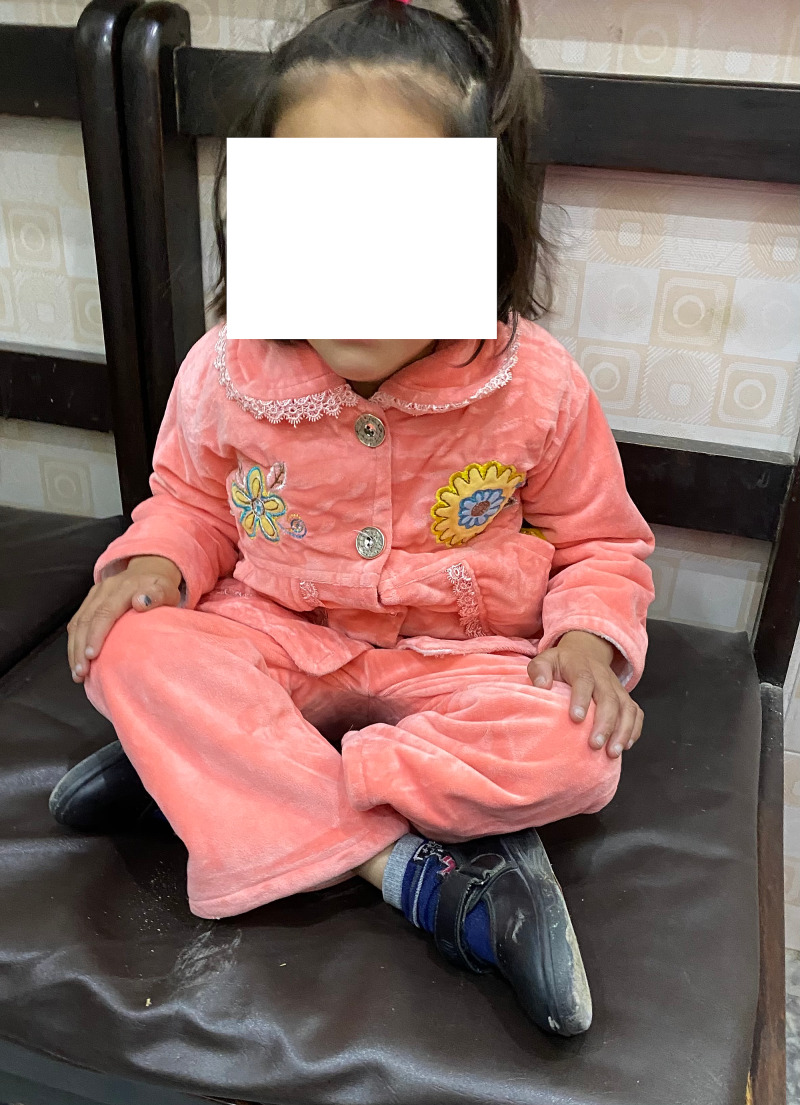
Post Salter's osteotomy, palthi I position on Bhatti's score

**Figure 14:**
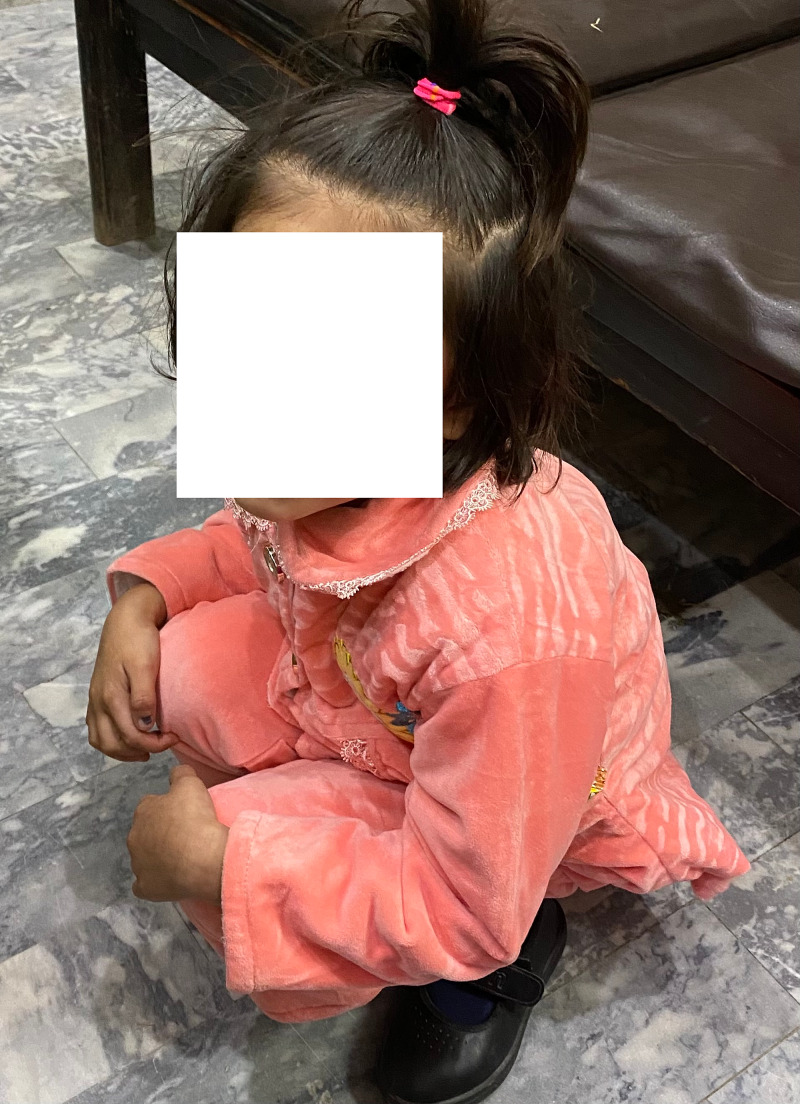
Post Salter's osteotomy; squat I position on Bhatti's Functional Scoring

**Figure 15:**
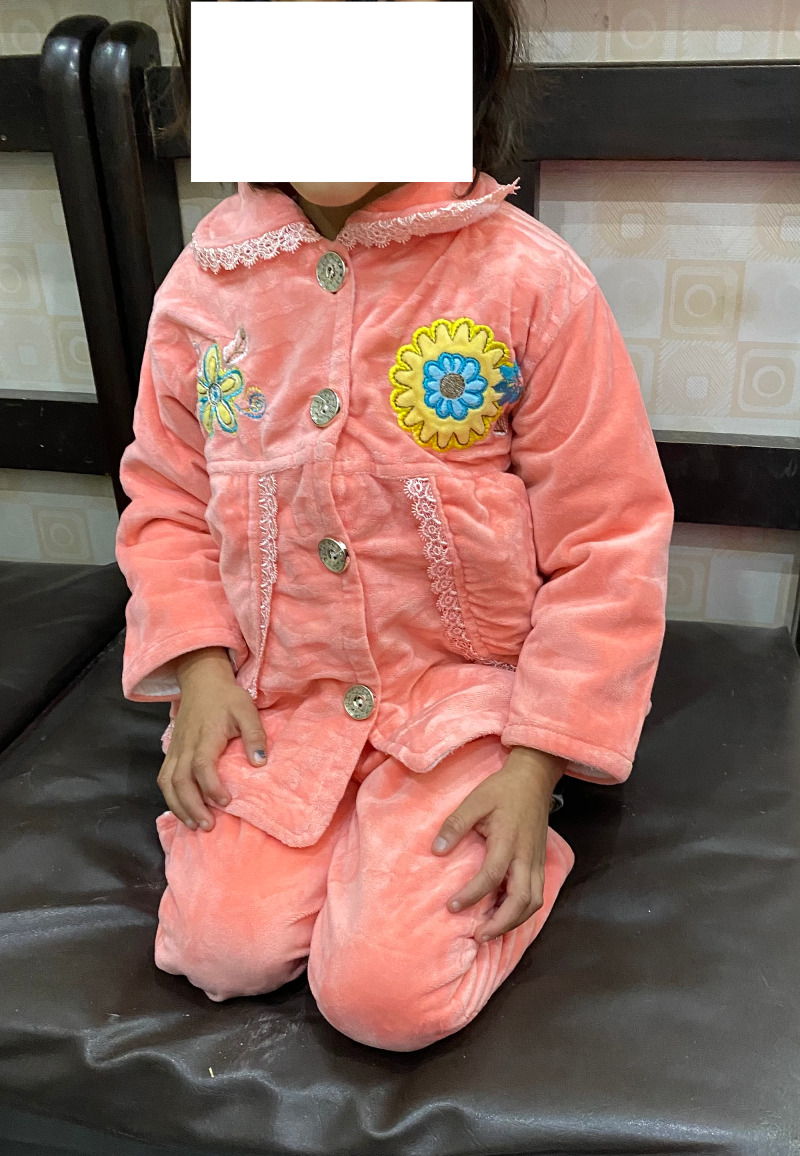
Post Salter's innominate osteotomy, tashahhud I position on Bhatti's score

On radiological evaluation with Severin classification, Table 5 indicates that the majority (85.36%, 70/82 hips) achieved satisfactory results with Class IAB and IIAB, i.e. normal and nearly normal hip joint development. While 12.19% (10) hips developed moderate (Class III) deformity of the caput femoris and 2.43% (two) hips developed mild (class IVA) to frank subluxation (Class IVB), including one unilateral and the other on the left side of a bilateral DDH. Whereas on the overall clinicoradiological outcome (Table 5), 84.28% (59/70) hips with Severin Class I and II behaved excellent to good, 15.71% (11/70) hips behaved fair, and there was no poor outcome. Out of 10 hips that scored Severin Class III, 60% (6/10) hips behaved excellently to good on BFSS and 40% (4/10) hips behaved fair on BFSS (Figures 7-15). On comparing the postoperative radiological outcome with BFSS using Fisher’s exact test, statistically, no significant difference was observed with p-value=0.803. Similarly, statistically, no significant difference was found between Severin class and age groups (p>0.05) and the association between outcome and gender, laterality, and Tonnis grade (p>0.05). In the seven hips in six patients of age below two years, two had open reduction along with distal derotation osteotomy; they achieved Severin Class I AB in four hips, Class III in two hips, and Class IVB in one hip. Clinically, on BFSS, they behaved satisfactorily in five and poor in one hip and an acetabular index mean of 24.57+/-SD2.89 (Tables 5-6). The outcome was similar to the Salter's group, whereas five hips in four patients in the age group over eight years achieved Severin Class I in two hips and Class II in three hips clinically on BFSS behaved satisfactorily (Fair). While the acetabular index decreased statistically significant (p=0.001), i.e. from mean 53 +/-SD4,47 to mean 23+/-SD 2.45 (Tables 4-5). The outcome was similar to Pemberton osteotomy with fair clinico-radiological results.

**Table 5:** Radiological outcome as per age groups, procedures, and comparison of Severin Class on Bhatti’s score (n:82 hips)

SEVERIN’S CLASS								
IA	IB	IIA	IIB	III	IVA	Lt. IVB	p-value	
Age groups									
1-3 years	7	19	3	12	6	1	1	0.19	
3.1-5 years	3	8	0	3	4	0	0		
5.1-8 years	2	5	0	2	0	0	0		
>8 years	3	0	0	3	0	0	0		
Procedure									
Dega + Shortening	2	0	0	2	0	0	0	0.819	
Open Reduction Capsulorrhaphy Unilateral	1	1	0	0	2	0	1		
Open Reduction Capsulorrhaphy ~ Bilateral	0	0	0	2	0	0	0		
Pemberton Unilateral	2	8	1	0	1	0	0		
Pemberton + Shortening	2	2	0	0	0	0	0		
Pemberton + Shortening ~ Bilateral	6	5	0	5	0	0	0		
Pemberton ~ Bilateral	0	3	0	0	1	0	0		
Salter Unilateral	0	4	0	4	0	1	0		
Salter + Shortening	0	5	0	1	1	0	0		
Salter + Shortening ~ Bilateral	1	3	1	3	2	0	0		
Salter ~ Bilateral	1	1	1	2	3	0	0		
Bhatti’s Score									
Poor	2	2	0	0	0	0	0		0.803	
Fair	2	7	0	6	3	0	0			
Good	3	9	0	4	2	1	0			
Excellent	11	17	3	13	5	0	1		

Tables 6-7 refer to the change in the acetabular index (AI) after surgery at a minimum follow-up of three years. The paired t-test was applied to assess the difference between pre- and post-treatment acetabular index. The mean acetabular index significantly decreased in Dega + shortening, Pemberton, Pemberton + shortening, Pemberton + shortening ~ bilateral, Pemberton ~ bilateral, Salter, Salter + shortening, Salter + shortening ~ bilateral, and Salter ~ Bilateral (p<0.05). Whereas no significant change was observed in the Pemberton ~ bilateral group (p>0.05). Comparing outcomes in both Pemberton's and Salter’s groups, the acetabular index significantly reduced after both procedures (p<0.05). However, the Pemberton group was more effective than the Salter group in reducing the acetabular index. On progressive development of the hip with a three to six-year of follow-up, patients with Salter’s osteotomy exhibit a stationary acetabular index as achieved at the three months post-operative follow-up and thereafter. Whereas, in patients with Pemberton osteotomy, the acetabular exhibited a decreasing trend in the acetabular index (Pemberton 10.1%, Salter 4.3%). The finding suggests a progressive radiological improvement in anterior acetabular coverage after Pemberton’s acetabuloplasty as compared to the Salter’s group.

**Table 6:** Mean change in the acetabular index before and after treatment

		Pre		Post		Change		p-value
	n	Mean	SD	Mean	SD	Mean	SD	
Overall	82	42.41	7.40	23.49	3.67	18.93	7.52	0.001
Operations								
Dega + Shortening	5	53.00	4.47	23.00	2.45	30.00	5.10	0.001
Lt. Open Reduction Capsulorrhaphy + Distal Derotation Osteotomy	1	35.00	-	22.00	-	-	-	-
Open Reduction + Distal Derotation Osteotomy	1	40.00	-	22.00	-	-	-	-
Open Reduction Capsuloraphy	3	31.67	2.89	26.67	2.89			
Open Reduction Capsuloraphy ~ Bilateral	2	35.00	-	25.00	-	-	-	-
Pemberton	11	42.73	4.67	20.55	2.88	22.18	3.66	0.001
Pemberton + Shortening	4	47.50	1.44	24.00	2.94	23.50	3.11	0.001
Pemberton + Shortening ~ Bilateral	16	40.63	5.44	20.63	1.78	20.00	4.20	0.001
Pemberton ~ Bilateral	4	32.50	14.43	22.75	2.22	9.75	16.48	0.322
Rt. Pemberton	1	40.00	-	20.00	-	-	-	-
Salter	8	39.13	7.86	25.38	1.69	13.75	7.36	0.001
Salter + Distal Derotation Osteotomy	1	40.00	-	23.00	-	-	-	-
Salter + Shortening	7	45.00	6.45	25.29	2.14	19.71	6.42	0.001
Salter + Shortening ~ Bilateral	10	48.00	2.58	26.00	2.54	22.00	3.77	0.001
Salter ~ Bilateral	8	43.13	5.30	26.38	5.73	16.75	5.01	0.001

**Table 7:** Pre and post-treatment acetabular index in Pemberton's versus Salter's group

Groups	n	Acetabular index				Change		p-value
		Pre		Post				
		Mean	SD	Mean	SD	Mean	SD	
Pemberton group	36	41.11	7.18	21.19	2.94	19.92	7.10	0.001
Salter plus group	34	44.03	6.38	25.79	3.27	18.24	6.29	0.001

Table 8 refers to the development of complications that reveals avascular necrosis (AVN) of the caput femoris in 9.75% (8) hips in six patients. One of these AVN was associated with subluxation (Class IVA), which was noticed at four months postoperative. The patient was three years old at the time of surgery. Another patient had subluxation IVB on one side of bilateral DDH of an 18-month-old, which was noticed at nine months postoperative. Both these cases of subluxation and AVN had Salters osteotomy. Ten hips (12.19%) in seven patients, who were below five years at the time of the surgery, had significant stiffness and impingement. One of them was due to postoperative sepsis in both sides of bilateral DDH. All these patients had a score fair on BFFS. Two of them aged >8 years were operated on with Dega’s osteotomy. Salter’s group had total AVN in four hips and Pemberton's group had AVN in two hips of a bilateral DDH. Subluxation was seen in Salter and not in Pemberton. Whereas impingement and stiffness were more in the Pemberton and Dega group but not in the Salter group.

**Table 8:** Postoperative complications with various procedures AVN: avascular necrosis; DFDRO: distal femoral derotation osteotomy

Procedures	Open reduction + capsulorrhaphy			Open reduction + pelvic osteotomy		Open reduction + femoral shortening + pelvic osteotomy			Hips N=82	Patients N=60
	OR	DFDRO	PO	Salter	Pemberton	Salter	Pemberton	Dega		
AVN (Kalamchi =K)	-	KI=1 KII=1	-	K1=1	-	KIII=3	KIII=1 KIV=1	-	8 (9.75.%)	6
Subluxation (Severin’s IVA, B) One each	-	-	-	1	-	1	-	-	2 (2.47%)	2
Wound Infection	-	-	-	-	-	-	2	-	2 (2.47%)	1
Stiff Hip and Impingement	-	-	-	-	3	-	5	2	10 (12.19%)	7

## Discussion

The overall clinical outcome of BFSS in unilateral cases was much better than bilateral DDH (Table 3). The majority 21/22 (95.45%) of bilateral DDH patients were operated on in a single go, under the same setup, and were immobilized in a cast for the same duration as unilateral cases. The obvious reason may be the double leg morbidity vs unilateral. With unilateral DDH, the free leg usually supports early recovery in the range of motion (ROM) exercises and weight-bearing. Besides less satisfactory clinical outcome on BFSS amongst bilateral vs unilateral DDH, we found one of the hips in bilateral DDH often has a complication of either subluxation or AVN (Table 8) [12-14]. Karol has also identified "bilateral DDH as the only poor prognostic factor” [18]. On comparing the functional results of procedures on BFSS (Table 2 and Table 4), Salter’s group behaved better than Pemberton's amongst both intragroup and intergroup outcomes. Patients with distal femoral derotation osteotomy (DFDRO) did not produce any adverse effect in the limitations of ROM at the knee and the development of angular deformity. Moreover, on functional outcomes, they behaved similarly to Salter’s group on BFSS.

On the overall radiological evaluation (Table 5), the 85.36% (70/82) hips achieved satisfactory results with Severin Class IAB to Class IIAB, with a nearly normal hip joint development. Amongst them, 84.28% (59/70 hips) behaved excellently to good on BFSS. However, on comparing the individual postoperative radiological score with Bhatti’s scoring system using Fisher’s exact test, no statistically significant difference was observed, with p-value=0.803 (Table 4). Similar to Ezirmik, Kadri, and Bhuyan, the current study observed a better ROM outcome in the SIO group than the PPO group [19-20]. They cited more lateral translation in the PPO group than SIO, as the case may be. The current study also supports the observation of Ezirmik and Kadri that “PPO provides better coverage and stability, as it is an incomplete osteotomy compared to the SIO group and that PPO does not need a trans-fixation K wire stabilization” [19].

On the intra and intergroup assessment of functional results on BFSS in various age groups at the open reduction, we noticed a progressive decline in clinicoradiological outcomes, as the age increases over three to five years and thereafter (Tables 3-5). This observation supports the citation of Zadeh et al. and Okano et al. that “on long-term followup, the age at operation has been the significant prognostic factor” and “the acceptable radiological results of 94% at the age of two years decline to 80% when the procedure was undertaken between two and four years and to 71% when aged over four years” [4,21].

The PPO has been cited as a suitable option for severe dysplastic acetabulum, subluxation, and in older children to prevent redislocation [4]. However, PPO produces more AVN and impingements, alteration shape, and capacity of the hip joint as compared to the SIO group [1,19-20]. Whereas, in the current study, despite the higher rate of anterior impingement and stiffness noticed in Pemberton’s group, paradoxically, AVN was less, i.e. 2.43% (2/82 hips) in PPO than in the SIO group, i.e. 4.87% (4/82). A similar observation was cited by Ezirmik and Kadri who reported AVNs in 31% (5/16 hips) in PPO as compared to more AVNs, i.e. 68.75% (11/16 hips) in Salter’s group [19], whereas Bhuyan reported only 4% (1/25) AVN with SIO group [20]. Ezirmik and Kadri further reported coxa magna and coxa vara rates to be twice more common in the SIO group as compared to PPO [19]. However, such a late complication of coxa valga, vara, and brevia were not yet seen in the current study with follow-up (3-6 years), as that requires a longer follow-up duration [4,18-23]. We find a minimum risk of bleeding, sciatic nerve damage, and structural alteration of the curvatures and volume of the pelvis with PPO, as reported by Ezirmik, Kadri, and Bhuyan et al. [19-20].

The AI in DDH is reported as a reasonable measure in children younger than eight years. Whereas the center-edge (CE) angle becomes useful only in the patient who is more than 5 years of age and is most useful in adult patients [24]. Since our patients were of both age ranges, thus we have used both these parameters in this study as the parameter for radiological assessment (Table 5). Ibrahim et al. in their study on Turkish children have referred to the mean acetabular index of males in the age group of one and eight years as ranging from 21.0 degrees to 13.8 degrees, with the lowest limit mean from 23.6 to 14.2 degrees [17]. Whereas in females, the mean acetabular index is in the same age group, ranging from 23.6 degrees to 14.2 degrees and the lowest is 33 degrees to 23 degrees. We found a nearly similar acetabular index on the normal side of our 38 unilateral cases, i.e. within the range of 25-15 degrees. Hence, we used this range as a baseline comparative indicator for postoperative evaluation in unilateral as well as bilateral cases. The investigations by Salter and Begatur report the mean acetabular index with SIO as 10 to 23.5 degrees [2,25]. Whereas Bhuyan, Kessler, and Zrore reported the achievement of an acetabular angle of 5 to 35 degrees with PPO [20,26-27]. The current study achieved AI within the range reported by Salter, Bhuyan, Begatur, Kessler, and Zrore and nearly similar to Ezirmik's findings [2,19-20,25-27]. Ezirmik and Yildiz reported the mean correction degree of the AI to be better with PPO than SIO [19]. Similar to the findings of Ezirmik and Yildiz, we also achieved a mean CE angle of Weiberg CE as 37.15 degrees with SIO and 43.11 degrees with PPO [19]. Thus, the overall improvement in the acetabular index and CE angle in this study significantly reduced and was better with PPO as compared to SIO (Tables 6-7). The acetabular coverage progressively improved as the child grows, along with a decrease in irregularity seen on the earliest postoperative follow-up, whereas the acetabular index remained stationary as achieved at earlier follow-up in the SIO group [1,4,18].

Most of our PPO group patients had an acetabular irregularity, ill-defined joint line, sclerosis, and ill-defined cartilage space as cited by McKay but none in the SIO group. McKay cited AVN of acetabular cartilage or growth disturbance as a cause of these changes. He further observed none of these changes occurs with Iliopsoas tenotomy and transfer to the anterior capsule [1]. These irregularities of the acetabulum gradually diminish and lead to the development of congruent hips over the years follow-up. The literature supports this phenomenon of spontaneous resolution of dysplastic features and the development of a normal hip, due to the mutual growth-stimulating effect of concentric anatomical reduction without tightness [1,4,10,18-19]. Functionally, on BFSS, these patients behaved better soon after the removal of the cast, with good rehabilitation exercises, especially in the younger than five years age group at the time of surgery [4,18].

All the AVNs in Salter’s group in the current study (Table 8) were noticed in the age group less than three years, though it has been cited more amongst the age group over three years [4,18]. The cause may be the post-reduction tightness of the hip following SIO and no femoral shortening performed, as the age of the patient was < 18 months. The other reasons may be the extensive inferomedial capsular dissection, tight capsulorrhaphy, which has also been cited by multiple investigators, who further add to the list of reasons as being the perioperative microvascular insult, tight posterior capsule, and eccentric posturing of the femoral head in plaster [4,21,28-30]. Furthermore, Zadeh et al. observe the position of excessive abduction, internal rotation, and tight reduction as a cause of vascular disturbance and premature physeal fusion that further led to late complications of the coxa valga, vara, and brevia on long-term follow-up [4,18,28]. In this study, nothing similar happened, as all the cases were immobilized in the neutral position of weight-bearing, meticulously dissected, with liberal use of femoral shortening in the age group over 30 months. To minimize the risk of AVN, McKay reported that rerouting the Iliopsoas tendon after tenotomy, under the straight head of the rectus, and reattaching to the anterior capsule shall achieve a stable concentric reduction with a minimize pressure over the femoral head and, subsequently, lower the risk of AVN [1].

In the patient with mild subluxation (Severin Class IVA), it was due to Kalamchi III AVN. This patient responded well with non-weight-bearing mobilization exercises and Craig splint for the next two months. She developed a moderate deformity of the hip (Severin Class III) with the last follow-up of five years. Similarly, the hip that developed Severin Class IVB subluxation also responded to fair (Severin Class II) after a re-do femoral de-rotation osteotomy as cited by MacKay as well [1]. Her acetabular index improved from 50 degrees to 25 degrees on the right side and 380 on the AVN side. She clinically behaved good on BFSS on that side and excellent on the other side at the five-year nine-month follow-up. Whereas our patients who had bilateral surgery done after the age of six years, unfortunately, developed skin-deep wound infection, along with AVN type III on one and AVN type IV on the other side. Her sepsis gets settled with multiple dressings and antibiotics as per the culture and sensitive report. She developed significant stiffness till the last follow-up at over three years.

The stiffness observed in this series was mostly due to the non-availability of a rehabilitation program, as the majority of our patients came from the countryside, from a far-flung northern area (1500-1800 kilometers away). Some negligence on the part of parents who lives in major cities but could not afford time and finance to get their children rehabilitated well in time. That too produces a significant limitation in the management of our cases. 

Zadeh et al. support the position on neutral weight-bearing after open reduction as of paramount importance if a satisfactory long-term outcome is to be achieved. The benefits of this position of immobilization include decreased duration immobilization to six to eight weeks, no need for an orthosis, early in-bed mobilization, and rapid recovery to regain weight-bearing exercises and mobilization [4]. Hence, we used spica cast immobilization for a minimum of six weeks and allow non-weight-bearing exercises soon after the removal of the cast and weight-bearing within 10 weeks. Bhuyan also recommends the same duration of six weeks but a further four weeks in an abduction splint; that splint, we feel, was not necessary, as non-weight-bearing mobilization significantly helps in reducing postoperative stiffness [16,20].

The limitation of this study was the mid-term follow-up duration (three to six years), as the radiological outcome has been reported as deteriorated in a significant number of cases during adolescent growth spurts and thereafter till 30 years, mainly because of premature physeal arrest [4,18,21,23]. Similar development of late complications of the coxa magna, vara, and valga was observed in a few of our published cases in 1997, 2009, and 2014. These too were mostly in patients who developed AVN, postoperative sepsis, and subluxation Severin Class IVA [12-14].

## Conclusions

We conclude that, on a medium-term follow-up duration of three to six years, the hips with PPO exhibited better acetabular coverage and progressive development of hips as compared to the SIO group. PPO is the best option in single-stage bilateral DDH open reductions, as it exhibits less bleeding, good stability, better postoperative pain control on mobilization, and a second surgery to remove transfixation K wires. This is contrary to other reports. Both groups, however, behaved equally on functional assessment and limitations in accustomed sitting habits as assessed with Bhatti’s Functional Score System. The risk of subluxation and AVN was found a little higher in the SIO group, Whereas there was femoroacetabular impingement in the PPO group. The progressive hip development and clinical evaluation on BFSS in the younger than three years group was better than in the over three years age group. The preoperative radiologic evaluation and per-operative assessment with Catterall’s test of stability were found to be a better tool for proper judgment to have additional osteotomies of the femur and pelvic to get better coverage of the caput femoris.
